# Correlates of Covid-19 Vaccine Acceptance among Residents of Ohio: A Cross-sectional Study

**DOI:** 10.1186/s12889-022-12661-8

**Published:** 2022-02-04

**Authors:** Zelalem T. Haile, Anirudh Ruhil, Benjamin R. Bates, Orman Hall, Mario J. Grijalva

**Affiliations:** 1 Department of Social Medicine, Ohio University Heritage College of Osteopathic Medicine, Dublin, OH 43016 USA; 2 Infectious & Tropical Disease Institute, Ohio University, Athens, OH USA; 3 Voinovich School of Leadership and Public Affairs, Ohio University, Athens, OH USA; 4 School of Communication Studies, Ohio University, Athens, OH USA; 5 College of Health Sciences & Professions, Ohio University, Athens, OH USA; 6 Department of Biomedical Sciences, Ohio University Heritage College of Osteopathic Medicine, Athens, OH USA

**Keywords:** COVID-19 vaccine, vaccine acceptance, vaccine hesitancy, public health, prevention

## Abstract

**Background:**

Recent studies in the United States have shown that between 56 to 74% are willing to receive the COVID-19 vaccine. A significant portion of the population should be vaccinated to avoid severe illness and prevent unnecessary deaths. We examined correlates of COVID-19 vaccine acceptance among a representative sample of adults residing in Ohio.

**Methods:**

We conducted a cross-sectional study using an online platform (*n* = 2358). Descriptive statistics, chi-square test and multivariable regression analysis were performed.

**Results:**

Overall, 59.1% of the participants indicated COVID-19 vaccine acceptance to be vaccinated. In the multivariable model, the likelihood of COVID-19 vaccine acceptance was lower for younger individuals compared to those 55 years and older. The odds of COVID-19 vaccine acceptance were lower for: females compared to males (OR 0.58, 95% CI: 0.47–0.71; *P* = 0.001), non-Hispanic blacks compared to non-Hispanic whites (OR: 0.49 95% CI: 0.35–0.70; *P* = 0.001), previously married (OR 0.64 95% CI: 0.49–0.84; *P* = 0.002) and never been married (OR 0.75 95% CI: 0.59–0.96; *P* = 0.023) compared to married people, individuals with less than high school (OR 0.21 95% CI: 0.08–0.60; *P* = 0.003) and high school education (OR: 0.45 95% CI: 0.36–0.55; *P* < 0.001) compared to those with education beyond high school, and for individuals who had no confidence in the abilities of the state government (OR 0.69 95% CI: 0.53–0.89; *P* = 0.005) and other world governments to combat COVID-19 (OR 0.67 95% CI: 0.50–0.91; *P* = 0.009). A one unit increase in knowledge about COVID-19 (OR 1.19, 95% CI: 1.13–1.26; *P* < 0.001), behavioral adherence (OR 1.25, 95% CI: 1.15–1.37; *P* < 0.001), perceived susceptibility (OR 1.10, 95% CI: 1.03–1.17; *P* = 0.004), perceived severity (OR 1.09, 95% CI: 1.03–1.16; *P* = 0.003), and trust in COVID-19 messages from the government scores (OR 1.08, 95% CI: 1.06–1.10; *P* < 0.001) were associated with an increase in the likelihood of COVID-19 vaccine acceptance.

**Conclusions:**

COVID-19 vaccine acceptance differed by sociodemographic and other modifiable factors. Findings can inform local public health authorities in the development of effective, context-specific communication strategies to improve vaccination uptake.

**Supplementary Information:**

The online version contains supplementary material available at 10.1186/s12889-022-12661-8.

## Background

Globally, over 5.3 million people have died of COVID-19 (as of December 21, 2021) [1]. The United States shares 799,942 of these deaths. In Ohio, over 1.8 people had COVID-19 and 28,277 people have died of COVID-19 [2]. Several preventive strategies including the use of masks, hand hygiene, physical distancing, contact tracing, screening, quarantining and isolating have both been promoted and institutionalized to prevent COVID-19 infection [3, 4]. Additionally, the advent of safe and effective COVID-19 vaccines has become the most crucial long-term solution to prevent further morbidity and mortality in the population [5, 6].

At least seven COVID-19 vaccines have been approved and rolled out in various countries [7]. In the United States, three COVID-19 vaccines are currently authorized (as of March 4, 2021) and trials are in progress for two additional vaccines [8]. While this is a major public health achievement, it is important for a significant portion of the population to be vaccinated to avoid severe illness and recurrence of COVID-19 and prevent unnecessary deaths. Unfortunately, however, antivaccine movements in the US and elsewhere have slowed vaccine uptake in many communities, especially in countries where mandatory vaccination is not the law or infeasible because of political and other considerations [9–13]. These movements use false or misleading information to not only discourage individuals from getting vaccinated but also encourage them to violate basic preventive behaviors such as mask wearing, adhering to stay-at-home orders, and participating in contact tracing initiatives [9, 14]. Additionally, concerns with the necessity, safety and potential side effects, trust in information from government sources, perceived risk of COVID-19 and COVID-19 vaccine, trust in the medical industry including pharmaceutical companies involved in the vaccine development have been identified as reasons for being unsure or unwilling be vaccinated [15–18].

The success of mass vaccination programs is determined by coverage and willingness to get vaccinated. The SAGE Working Group on Vaccine Hesitancy defined vaccine hesitancy as the delay in acceptance or refusal of vaccination despite availability of vaccination services [19]. Recent studies in the US have shown that between 56 to 74% are willing to receive the COVID-19 vaccine [20–23]. However, acceptance rates are lower for certain sociodemographic groups including blacks, Hispanics, persons living in rural areas, and individuals with lower levels of educational attainment [20, 21, 23]. When COVID-19 vaccines became available each state followed its own phase-based vaccination plan to determine vaccination eligibility. Initially, in almost every state, priorities were given to healthcare workers and residents of long-term care facilities based on the recommendation from the Advisory Committee on Immunization Practices [20]. Ohio was one of the few states that made COVID-19 vaccination eligibility before April 2021 when every state had made all adults 18 and older eligible to get vaccinated. At present, 5–11 years old and all teens can get the Pfizer-BioNTech vaccine [24]. As of December 17, 2021 only 54.5% of Ohioans were fully vaccinated [25]. In addition to national trends, since each state is charged with rolling out COVID-19 vaccines, identifying factors associated with COVID-19 vaccine acceptance in each state can assist local public health authorities in the development of effective, context-specific communication strategies to improve vaccination uptake. The objective of this study was to identify correlates of COVID-19 vaccine acceptance among a representative sample of adults residing in Ohio. We hypothesize that sociodemographic characteristics would be associated with COVID-19 vaccine acceptance. We further hypothesize that the association between sociodemographic factors and COVID-19 vaccine acceptance would vary by region of residence.

## Methods

### Study design

A self-administered cross-sectional survey was conducted from January 14 to 22, 2021. Eligibility criteria included being age 18 or older and currently residing in Ohio. Participants were recruited using an online survey panel accessed through Qualtrics (Provo, UT). Qualtrics.

maintains dedicated Research Panels primarily of adults who have agreed to participate in surveys for a nominal fee. These panel members have known demographics including geographic location that can be tapped for simple random or stratum-based sampling schemes. Members of the Qualtrics panel were invited by email to complete the questionnaire online. First, participant eligibility was determined using a brief online screener. Eligible participants were then provided an informed consent notification prior to their completing the online survey. A total of 2358 individuals both consented to and completed the survey. To maintain representativeness, the gender, age, race/ethnicity, and regional distributions of the sample were selected to represent of the distribution of these characteristics in Ohio. Participants were provided an incentive for participation from Qualtrics. Data provided to the researchers were fully de-identified. The study was deemed exempt by the Institutional Review Board of the authors’ institution (#20-E-281).

### Measures

#### Outcome of interest: COVID-19 vaccine acceptance

We assessed COVID-19 vaccine acceptance based on how strongly participants agreed with the following statement measured on a 5-point Likert scale (1 = strongly disagree to 5 = strongly agree): “*If a vaccine becomes available and is recommended for me, I would get it.*” Based on existing literature [21, 26, 27], this variable was dichotomized to create a vaccine acceptance indicator no (0 = strongly disagree/disagree/neither agree nor disagree); yes (1 = Strongly agree/agree).

#### Explanatory variables

##### Sociodemographic characteristics

Participant characteristics captured in the survey included age, gender, race/ethnicity, marital status, educational attainment, employment, and geographic region.

##### Knowledge, attitude, and practice

Knowledge of COVID-19 was assessed by calculating of the sum of twelve knowledge items covering signs and symptoms, transmission routes, prevention practices. Response options include yes, no and do not know. This measure has been used previously by Zhong et al.’s [28] was later modified by Bates et al. [29, 30]. We created a knowledge score by assigning one point for each question answered correctly, resulting in a total score of 0–12 correct responses. Perceived severity of contracting COVID-19 was measured using two items on a 5-point Likert scale ranging from 0 (strongly disagree) to 4 (strongly agree) scale. Participants responded to statement “*If I were to contract COVID-19, the consequences would be very severe*” and “*If I were to contract COVID-19, I would become very ill*.” These items were summed to create a composite score ranging from 0 to 8 with higher scores indicating higher level of perceived severity. Perceived susceptibility was measured using two items on a 5-point Likert scale ranging from 0 (strongly disagree) to 4 (strongly agree) scale: “*I am likely to contract COVID-19*” and “*People like me are vulnerable to COVID-19*.” We summed these items to create a composite score ranging from 0 to 8 with higher scores indicating higher level of perceived susceptibility. Behavioral adherence was measured by asking participants five questions regarding COVID-19 preventive behaviors on a dichotomous scale (yes/no). These include *avoiding social gatherings, avoiding family gatherings, avoiding crowded places, wearing a mask, and regular handwashing*. For each preventive behavior, participants were assigned a score of 1 if they were adherent and 0 if they were non-adherent. Items were summed to create a composite score ranging from 0 to 5, with higher scores indicating higher level of adherence to the recommended preventive behaviors.

##### Confidence in government

Confidence in the government’s abilities to combat COVID-19 were measured using three items on a dichotomous (yes/no) scale. Participants indicated their agreement that they were confident in the state government, the US government, and other governments around the world to successfully respond to COVID-19.

##### Trust in COVID-19 messages from the government

Trust in government as a messenger was measured using items on a five-point measure a 0 (strongly feel this way) to 4 (strongly feel other way) scale. A common stem “*Generally, messages from the government about COVID-19 are …*” was employed with six-word pairs: accurate-inaccurate, correct-incorrect, trustworthy-untrustworthy, false-true [reverse-coded], ethical-unethical, and generous-selfish. The Cronbach’s alpha coefficient for these items was 0.88, suggesting that the items have relatively high internal consistency. Items were summed to create a composite score ranging from 0 to 24, with higher scores indicating greater trust in government messages about COVID-19.

### Statistical analysis

Descriptive statistics including mean, standard deviation, frequencies, and proportions were employed to summarize and describe data. To compare differences in vaccine acceptance by participants’ characteristics, we utilized Chi-square tests for categorical variables and T-tests for continuous variables. Unadjusted and multivariable-adjusted logistic models were fitted to estimate the crude odds ratio (OR), adjusted odds ratio (aOR), 95% confidence interval (CI) for odds ratios, for each of the independent explanatory variables. All variables with *p* < 0.2 in the unadjusted analyses were included in the multivariable-adjusted model. A two tailed *p*-value < 0.05 was considered statistically significant. We assessed potential collinearity among the independent variables using variance inflation factor (VIF) > 4. The Hosmer–Lemeshow goodness of fit test was used to assess model goodness of fit. To test the hypothesis that the association between sociodemographic factors and COVID-19 vaccine acceptance would vary by region of residence, a stratified analysis by region was performed. Finally, to address potential concerns regarding residual confounding we conducted sensitivity analysis by excluding participants who responded neither agree nor disagree to the question on COVID-19 vaccine acceptance. However, we did not conduct stratified analysis by region for this subgroup because of the reduced analytic sample size that threatens the validity of results with this subgroup. All analyses were carried out using SAS 9.4 (SAS Institute, Inc., Cary, NC).

## Results

Descriptive statistics of the study population by COVID-19 vaccine acceptance are presented in Table 1. The mean age of the study population was 44.7 (standard deviation 16.66) years and 51.1% were female. A greater proportion of the participants were 55 years and older, were predominantly non-Hispanic white, were married, had attained high school education, were employed, were from metropolitan areas and reported that they are confident in the abilities of state and federal government, and other governments around the world abilities to combat COVID-19. Participants had high levels of knowledge about COVID-19 and behavioral adherence and moderate levels of perceived susceptibility, perceived severity, and trust in COVID-19 message from government.

**Table 1:** Characteristics of the study sample by COVID-19 vaccine acceptance (*n* = 2358)

	Overall	COVID-19 vaccine acceptance		
		No or Unsure	Yes	*p*^††^
	n (%)	n (%)	n (%)	
**Age (years)** (Mean 44.72 SD 16.66)				< 0.001
18–24	325 (13.8)	181 (55.7)	144 (44.3)	
25–34	442 (18.7)	195 (44.1)	247 (55.9)	
35–44	465 (19.7)	212 (45.6)	253 (54.4)	
45–54	393 (16.7)	175 (44.5)	218 (55.5)	
55+	733 (31.1)	202 (27.6)	531 (72.4)	
**Gender**				< 0.001^†^
Female	1205 (51.1)	552 (45.8)	653 (54.2)	
Male	1147 (48.6)	413 (36.0)	734 (64.0)	
Other	6 (0.3)	0 (0.0)	6 (100.0)	
**Race/ethnicity**				< 0.001
Non-Hispanic white	1952 (82.8)	761 (39.0)	1191 (61.0)	
Non-Hispanic black	201 (8.5)	110 (54.7)	91 (45.3)	
Hispanic or Latino	93 (3.9)	48 (51.6)	45 (48.4)	
Other	112 (4.8)	46 (41.1)	66 (58.9)	
**Marital status**				< 0.001
Never married	844 (35.8)	415 (49.2)	429 (50.8)	
Married	1097 (46.5)	360 (32.8)	737 (67.2)	
Separated/Divorced/Widowed	417 (17.7)	190 (45.6)	227 (54.4)	
**Education**				< 0.001
< High school	23 (1.0)	16 (69.6)	7 (30.4)	
High school	1281 (54.3)	640 (50.0)	641 (50.0)	
> High school	1054 (44.7)	309 (29.3)	745 (70.7)	
**Employment**				0.108
Unemployed	661 (28)	258 (39.0)	403 (61.0)	
Housewife/house husband	217 (9.2)	91 (41.9)	126 (58.1)	
Student	98 (4.2)	51 (52.0)	47 (48.0)	
Employed	1382 (58.6)	565 (40.9)	817 (59.1)	
**Region**				0.030
Appalachia South	201 (8.5)	97 (48.3)	104 (51.7)	
Appalachia North	174 (7.4)	79 (45.4)	95 (54.6)	
Non-Appalachia Rural	291 (12.3)	125 (43.0)	166 (57.0)	
Suburban	412 (17.5)	175 (42.5)	237 (57.5)	
Metro	1280 (54.3)	489 (38.2)	791 (61.8)	
**Confidence-state government**				< 0.001
No	841 (35.7)	463 (55.1)	378 (44.9)	
Yes	1517 (64.3)	502 (33.1)	1015 (66.9)	
**Confidence-federal government**				< 0.001
No	903 (38.3)	486 (53.8)	417 (46.2)	
Yes	1455 (61.7)	479 (32.9)	976 (67.1)	
**Confidence- governments around the world**				< 0.001
No	361 (15.3)	228 (63.2)	133 (36.8)	
Yes	1997 (84.7)	737 (36.9)	1260 63.1)	
**COVID-19 vaccine acceptance**				
No	965 (40.9)			
Yes	1393 (59.1)			
	Mean (SD)	Mean (SD)	Mean (SD)	p^‡^
**Knowledge about COVID-19** (Range 0–12)	9.29 (2.03)	8.63 (2.45)	9.75 (1.53)	< 0.001
**Behavioral adherence** (Range 0–5)	4.08 (1.19)	3.79 (1.36)	4.28 (1.00)	< 0.001
**Perceived susceptibility** (Range 0–8)	4.17 (1.90)	3.74 (1.93)	4.47 (1.81)	< 0.001
**Perceived severity** (Range 0–8)	4.18 (2.10)	3.71 (2.12)	4.51 (2.03)	< 0.001
**Trust in COVID-19 messages from the government** (0–24)	12.73 (5.49)	11.03 (5.26)	13.91 (5.34)	< 0.001

††Chi-square test *p*-values; † Fisher’s exact test *p*-value; ‡ Independent sample t-test *p*-value

Overall, 59.1% of participants (*n* = 1393) indicated COVID-19 vaccine acceptance and 40.9% (*n* = 965) indicated that they are not willing or unsure about getting vaccinated. Except for employment status, all other explanatory variables yielded statistically significant differences vis-à-vis COVID-19 vaccine acceptance. Participants who indicated they were not willing or unsure about getting vaccinated were more likely to be younger, female, non-Hispanic blacks, Hispanics, never married, had less than high school or high school education, were unemployed, lived in Southern Appalachia, or had no confidence in in the abilities of the state, federal, and other governments around the world to combat COVID-19. Compared to vaccine accepters, respondents not accepting vaccination had significantly lower mean scores on knowledge about COVID-19, behavioral adherence, perceived susceptibility, perceived severity, and trust in COVID-19 messages from the government (see Table 1).

In Table 2 we display the unadjusted and multivariable adjusted associations between characteristics of participants and COVID-19 vaccine acceptance. In the unadjusted model, all explanatory variables were significantly associated with COVID-19 vaccine acceptance. In the multivariable model, although the direction of the observed association persisted for all explanatory variables, the magnitude of effect declined. Compared to adults 55 years and older, the odds of COVID-19 vaccine acceptance were lower in all other age groups. Compared with males, the odds of COVID-19 vaccine acceptance were lower for females, with an adjusted odds ratio (95% confidence interval) of 0.58 (0.47, 0.71; *p* < 0.001). The odds of COVID-19 vaccine acceptance were lower for non-Hispanic blacks compared to non-Hispanic whites 0.49 (0.35, 0.70; *p* < 0.001). Compared to married people, the likelihood of COVID-19 vaccine acceptance was lower for previously married 0.64 (0.49, 0.84; *p* = 0.002) and those who have never been married 0.75 (0.59, 0.96; *p* = 0.023), respectively. Compared to those with education beyond high school, those with less than high school 0.21 (0.08, 0.60; *p* = 0.003) and high school 0.45 (0.36, 0.55; *p* < 0.001) education were less willing to accept the vaccine. The odds of COVID-19 vaccine acceptance were significantly lower for participants who had no confidence in in the abilities of the state government 0.69 (0.53, 0.89; *p* = 0.005) and other governments around the world to combat COVID-19 - 0.67 (0.50, 0.91; *p* = 0.009). Additionally, an increase in knowledge about COVID-19, behavioral adherence, perceived susceptibility perceived severity and trust in COVID-19 message from the government scores were positively associated with an increase in the likelihood of COVID-19 vaccine acceptance (see Table 2).

**Table 2:** Crude and multivariable-adjusted associations between characteristics of participants and COVID-19 vaccine acceptance (*n* = 2352)

	Crude OR (95% CI)	*p*	Adjusted OR (95% CI)	*p*
**Age**				
18–24	0.30 (0.23, 0.39)	< 0.001	0.67 (0.45, 1.00)	0.048
25–34	0.48 (0.38, 0.62)	< 0.001	0.66 (0.48, 0.91)	0.011
35–44	0.46 (0.36, 0.58)	< 0.001	0.51 (0.38, 0.69)	< 0.001
45–54	0.47 (0.36, 0.61)	< 0.001	0.57 (0.42, 0.77)	< 0.001
55+	Reference		Reference	
**Gender**				
Female	0.67 (0.56, 0.79)	< 0.001	0.58 (0.47, 0.71)	< 0.001
Male	Reference		Reference	
**Race/ethnicity**				
Non-Hispanic white	Reference		Reference	
Non-Hispanic black	0.53 (0.40, 0.71)	< 0.001	0.49 (0.35, 0.70)	< 0.001
Hispanic or Latino	0.60 (0.40, 0.91)	0.017	0.76 (0.47, 1.23)	0.263
Other	0.91 (0.62, 1.34)	0.621	0.90 (0.58, 1.40)	0.637
**Marital status**				
Never married	0.50 (0.42, 0.61)	< 0.001	0.75 (0.59, 0.96)	0.023
Married	Reference		Reference	
Separated/Divorced/Widowed	0.58 (0.46, 0.73)	< 0.001	0.64 (0.49, 0.84)	0.002
**Education**				
< High school	0.16 (0.06, 0.40)	< 0.001	0.21 (0.08, 0.6)	0.003
High school	0.42 (0.35, 0.49)	< 0.001	0.45 (0.36, 0.55)	< 0.001
> High school	Reference		Reference	
**Employment**				
Unemployed	Reference		Reference	
Housewife/house husband	0.88 (0.64, 1.20)	0.409	0.90 (0.62, 1.31)	0.583
Student	0.58 (0.38, 0.89)	0.013	1.16 (0.67, 1.99)	0.596
Employed	0.93 (0.77, 1.12)	0.448	0.88 (0.69, 1.12)	0.303
**Region**				
Appalachia South	0.66 (0.49, 0.89)	0.007	0.75 (0.53, 1.06)	0.104
Appalachian-North	0.75 (0.54, 1.03)	0.073	0.78 (0.54, 1.14)	0.195
Non-Appalachia Rural	0.83 (0.64, 1.07)	0.144	0.82 (0.61, 1.10)	0.188
Suburban	0.84 (0.67, 1.05)	0.125	0.79 (0.61, 1.03)	0.082
Metro	Reference		Reference	
**Confidence-state government**				
No	0.40 (0.34, 0.48)	< 0.001	0.69 (0.53, 0.89)	0.005
Yes	Reference		Reference	
**Confidence-federal government**				
No	0.42 (0.35, 0.50)	< 0.001	0.85 (0.65, 1.10)	0.204
Yes	Reference		Reference	
**Confidence- governments around the world**				
No	0.34 (0.27, 0.43)	< 0.001	0.67 (0.5, 0.91)	0.009
Yes	Reference		Reference	
**Knowledge about COVID-19**	1.35 (1.28, 1.42)	< 0.001	1.19 (1.13, 1.26)	< 0.001
**Behavioral adherence**	1.42 (1.32, 1.52)	< 0.001	1.25 (1.15, 1.37)	< 0.001
**Perceived susceptibility**	1.23 (1.18, 1.29)	< 0.001	1.10 (1.03, 1.17)	0.004
**Perceived severity**	1.21 (1.16, 1.26)	< 0.001	1.09 (1.03, 1.16)	0.003
**Trust in COVID-19 messages from the government**	1.11 (1.09, 1.12)	< 0.001	1.08 (1.06, 1.10)	< 0.001

*Abbreviations*: OR Odds ratio; *CI* Confidence interval

Due to small cell sizes participants who identify themselves other than male or female were excluded (*n* = 6)

Table 3 shows the association between associations between characteristics of participants and COVID-19 vaccine acceptance stratified by region. Overall, the results were consistent with the findings for the whole cohort. However, because of limited sample sizes and inadequate power, some of the Odds Ratios failed to reach conventional levels of statistical significance. In the sensitivity analysis excluding participants who neither agree nor disagree to the question on COVID-19 vaccine acceptance, results remained comparable to those reported in Table 2 (see Supplemental Table 1).

**Table 3:** Multivariable-adjusted associations between characteristics of participants and COVID-19 vaccine acceptance stratified by region

	Appalachia South OR (95% CI)	Appalachian-North OR (95% CI)	Non-Appalachia Rural OR (95% CI)	Suburban OR (95% CI)	Metro OR (95% CI)
**Age**					
18–24	0.59 (0.13, 2.62)	0.39 (0.08, 1.87)	0.18 (0.05, 0.61)**	0.90 (0.32, 2.56)	0.80 (0.46, 1.38)
25–34	0.43 (0.13, 1.39)	0.31 (0.08, 1.29)	0.38 (0.14, 1.06)	0.69 (0.30, 1.57)	0.87 (0.57, 1.33)
35–44	0.55 (0.21, 1.48)	0.28 (0.08, 1.04)	0.08 (0.03, 0.23)***	0.73 (0.35, 1.49)	0.61 (0.40, 0.91)*
45–54	0.92 (0.31, 2.74)	0.35 (0.12, 1.06)	0.20 (0.07, 0.57)**	0.60 (0.28, 1.29)	0.59 (0.39, 0.89)*
55+	Reference	Reference	Reference	Reference	Reference
**Gender**					
Female	0.44 (0.22, 0.91)*	0.42 (0.18, 0.97)*	0.53 (0.27, 1.03)	0.27 (0.16, 0.47)***	0.79 (0.60, 1.05)
Male	Reference	Reference	Reference	Reference	Reference
**Race/ethnicity**					
Non-Hispanic white	Reference	Reference	Reference	Reference	Reference
Other	0.78 (0.27, 2.26)	1.74 (0.42, 7.18)	1.18 (0.4, 3.46)	0.97 (0.47, 2.03)	0.49 (0.36, 0.66)***
**Marital status**					
Never married	0.59 (0.24, 1.47)	1.73 (0.6, 4.96)	0.38 (0.16, 0.88)*	0.69 (0.36, 1.33)	0.75 (0.54, 1.04)
Married	Reference	Reference	Reference	Reference	Reference
Separated/Divorced/Widowed	0.71 (0.29, 1.75)	0.47 (0.16, 1.34)	0.44 (0.17, 1.12)	0.71 (0.35, 1.44)	0.69 (0.47, 1.00)
**Education**					
≤ High school	0.32 (0.14, 0.69)**	0.24 (0.10, 0.60)**	0.38 (0.19, 0.77)**	0.42 (0.25, 0.72)**	0.47 (0.35, 0.62)***
> High school	Reference	Reference	Reference	Reference	Reference
**Employment**					
Unemployed	Reference	Reference	Reference	Reference	Reference
Housewife/house husband	0.54 (0.14, 2.03)	3.94 (0.88, 17.62)	1.16 (0.37, 3.65)	0.99 (0.39, 2.48)	0.73 (0.43, 1.24)
Student	2.25 (0.28, 17.81)	0.35 (0.05, 2.40)	0.49 (0.06, 4.41)	1.57 (0.41, 5.93)	1.57 (0.73, 3.36)
Employed	1.32 (0.58, 3.02)	1.95 (0.76, 5.00)	1.09 (0.49, 2.44)	0.53 (0.27, 1.04)	0.87 (0.62, 1.21)
**Confidence-state government**					
No	0.79 (0.26, 2.45)	0.32 (0.12, 0.86)	0.97 (0.42, 2.25)	0.58 (0.29, 1.13)	0.68 (0.48, 0.96)*
Yes	Reference	Reference	Reference	Reference	Reference
**Confidence-federal government**					
No	0.71 (0.24, 2.11)	0.71 (0.26, 1.93)	1.39 (0.57, 3.37)	0.72 (0.37, 1.42)	0.87 (0.62, 1.23)
Yes	Reference	Reference	Reference	Reference	Reference
**Confidence- governments around the world**					
No	0.50 (0.18, 1.38)	1.72 (0.59, 5.03)	0.37 (0.14, 1.01)	0.86 (0.39, 1.91)	0.62 (0.40, 0.94)*
Yes	Reference	Reference	Reference	Reference	Reference
**Knowledge about COVID-19**	1.11 (0.90, 1.36)	0.95 (0.75, 1.19)	1.25 (1.07, 1.45)**	1.32 (1.14, 1.53)***	1.23 (1.14, 1.32)***
**Behavioral adherence**	1.22 (0.92, 1.61)	1.90 (1.23, 2.94)	1.29 (0.98, 1.70)	1.16 (0.93, 1.46)	1.25 (1.10, 1.41)**
**Perceived susceptibility**	1.08 (0.87, 1.35)	0.94 (0.72, 1.22)	0.98 (0.80, 1.20)	1.35 (1.14, 1.61)**	1.09 (1.00, 1.19)*
**Perceived severity**	1.11 (0.92, 1.33)	1.20 (0.94, 1.54)	1.23 (1.01, 1.49)*	1.02 (0.87, 1.19)	1.09 (1.01, 1.18)*
**Trust in COVID-19 messages from the government**	1.01 (0.95, 1.07)	1.02 (0.95, 1.10)	1.20 (1.12, 1.28)***	1.10 (1.05, 1.16)***	1.08 (1.05, 1.11)***

*Abbreviations*: *OR* Odds ratio, *CI* Confidence interval; *p < 0.05, **p < 0.01, ***p < 0.001

Due to small sample sizes participants who identify themselves as non-Hispanic black, Hispanic/ Latino and other racial/ethnic groups were combined into “Other” group

## Discussion

In a large representative sample of Ohio residents, sociodemographic factors, knowledge, attitude, and practice related to COVID-19, confidence in government and trust in COVID-19 messages from the government were significantly associated with COVID-19 acceptance. The likelihood of COVID-19 vaccine acceptance was lower for younger, female, non-Hispanic blacks, never married or previously married people, those with less than high school or high school education, and those who have no confidence in in the abilities of the state government and other governments around the world to combat COVID-19. Also, an increase in knowledge about COVID-19, behavioral adherence, perceived susceptibility perceived severity and trust in COVID-19 messages from the government scores were associated with an increase in the likelihood of COVID-19 vaccine acceptance. As the COVID-19 vaccines continue to be rolled out, findings from our study could be used to target key demographic groups in the state who may resist to get vaccinated or have skepticism about the effectiveness of proven safety measures.

Lower likelihood of COVID-19 vaccine acceptance among younger individuals observed in the current study is consistent with other studies [20–22]. Younger individuals are significantly less likely to accept getting the vaccine compared with older adults, largely because they do not perceive the consequences of COVID-19 to be serious. While Ohio COVID-19 tracking data confirms that this cohort has minimal risk for severe consequences from contracting COVID-19, containing disease transmission will require widespread immunization of young Ohioans. In our study, women were significantly more likely to report vaccine resistance than men. This finding is also consistent with results from national surveys in the US [21, 22, 31]. Since women are often the most likely health gate keepers for two-parent households and are by default the gatekeepers for female headed households, they could have a disproportionate effect on the overall vaccination rate. Therefore, efforts could be undertaken to counter anti-vaccine disinformation directed at women.

In accordance with the present results, previous studies have demonstrated that non-Hispanic blacks were more likely to report lower likelihood of getting COVID-19 vaccination [20–23, 27, 32]. Potential explanations include lack of trust of the medical community that can be attributed to historical mistreatment of African Americans [33, 34], contemporary medical racism [35], everyday racism [36] and mistrust in systemic racism [37]. However, according to the most recent data racial disparities in vaccination rates have narrowed over time since the rollout of COVID-19 vaccination [38]. To assure health equity for all, the state could take steps to build trust by acknowledging their concerns and developing culturally tailored programs to educate minority populations about the safety and efficacy of COVID-19 vaccines. Most importantly, these programs should acknowledge the roots of mistrust in systemic racism and frame the discussion about distrust in in COVID-19 vaccines within the context of everyday racism and contemporary medical racism instead of past experiences of racism [35–37]. Our study revealed lower acceptance of getting vaccinated among never married and previously married individuals. To our knowledge, no prior studies have reported significant differences in the likelihood of vaccine acceptance by marital status. It is plausible that the observed associations can partially be explained by the fact that never married participants are more likely to be younger. Studies have also found that married individuals have better health experiences and outcomes compared to single or previously married individuals [39, 40].

Consistent with the literature, this research found that having a high school or less education reduces the likelihood of accepting COVID-19 vaccination [20, 22, 31, 32]. Lower educational attainment has been linked to greater levels concerns about riskiness and hesitancy towards new vaccines [41, 42]. Individuals with lower education are more likely to have lower health literacy and are more likely to accept antivaccination messages and may have low levels of trust of medical information. The state should give special consideration when discussing safety and effectiveness of COVID-19 vaccines with less educated individuals. Our finding of lower acceptance of getting COVID-19 vaccination among individuals who reported that they are not confident in the abilities of the state government and other governments around the world to combat COVID-19 is consistent with the previous literature [16]. Taken together, our findings indicate the need to build trust in the state government to improve COVID-19 vaccine uptake.

In our study, several modifiable factors that include knowledge about COVID-19, behavioral adherence, perceived susceptibility, perceived severity, and trust in COVID-19 messages from the government were positively associated with acceptance of a COVID-19 vaccination. Our findings are consistent with the previous studies that found similar results [27, 31, 32]. The modifiable nature of these factors makes them ideal candidates in the development of interventions aimed at improving knowledge, attitude and practice towards COVID-19 and improving uptake of COVID-19 vaccine. In an era where media and politics are influencing scientific decisions, it is important for the scientific community to find effective strategies to build confidence in evidence-based practices.

Several limitations should be considered. Firstly, the cross-sectional design precludes causal inferences. Secondly, the study was based on a convenience sample Qualtrics panel and may not have captured individuals who have no access to the Internet. However, the demographic characteristics of the participants in the study mirrors Ohio’s adult population. Thirdly, due to the nature of the data collection method, the study is susceptible to recall, self-report and social desirability biases. Fourthly, questions related to trust in COVID-19 messages from the government were not specific to state, federal or other governments around the world and respondents may have answered with different levels of government in mind. Finally, COVID-19 vaccination status of respondents was not captured. Although data for the current study were collected when COVID-19 vaccines were not widely available to the general public, it is possible that some participants were already vaccinated. The main strengths of the study include a large sample size, a sample representative of the state’s adult population, and the availability of several variables for adjustment in the multivariable model. Furthermore, unlike many studies of COVID-19 vaccination intentions conducted before or at the early stages of the development of the vaccine, our study was conducted after COVID-19 vaccines had started rolling out in the country but before Ohio started vaccinating the public (starting with those 75 years of age or older on January 25, 2021).

## Conclusions

In a large representative sample of Ohioan adults, COVID-19 vaccine acceptance differs by sociodemographic and other modifiable factors. We found lower likelihood of COVID-19 vaccine acceptance among younger, female, non-Hispanic black, never married or previously married, less than high school or high school education, and those who have no confidence in in the abilities of the state government and other governments around the world to combat COVID-19. Additionally, an increase in knowledge about COVID-19, behavioral adherence, perceived susceptibility perceived severity and trust in COVID-19 messages from the government scores were associated with an increase in the likelihood of COVID-19 vaccine acceptance. These findings highlight the need to develop targeted health promotion campaigns and programs aimed at improving COVID-19 vaccine uptake in the state.

## Supplementary Information

**Additional file 1: Supplemental Table 1.** Associations between characteristics of participants and COVID-19 vaccine acceptance excluding participants who responded neither agree nor disagree to get the COVID-19 vaccine (*n*=1941).

## Data Availability

The datasets used and/or analyzed during the current study are available from the corresponding author on reasonable request.

## Abbreviation

COVID-19: Coronavirus Disease 19
