# Supplementary material for: Correlates of Covid-19 Vaccine Acceptance among Residents of Ohio: A Cross-sectional Study
Source: BMC Public Health. 2022 Feb 4;22:226. doi: 10.1186/s12889-022-12661-8 (PMC8813184; doi:10.1186/s12889-022-12661-8)
Supplement: Supplementary file 1 — Additional file 1: Supplemental Table 1. Associations between characteristics of participants and COVID-19 vaccine acceptance excluding participants who responded neither agree nor disagree to get the COVID-19 vaccine (n=1941). [file 12889_2022_12661_MOESM1_ESM.docx]

| Supplemental table 1. Associations between characteristics of participants and COVID-19 vaccine acceptance excluding participants who responded neither agree nor disagree to get the COVID-19 vaccine (n=1941) | | | | | | |
| --- | --- | --- | --- | --- | --- | --- |
|  |  | Crude OR (95% CI) | p |  | Adjusted OR (95% CI) | p |
| **Age** | |  |  |  |  |  |
|  | 18-24 | 0.34 (0.24 , 0.46) | <0.001 |  | 0.79 (0.49 , 1.29) | 0.350 |
|  | 25-34 | 0.50 (0.37 , 0.68) | <0.001 |  | 0.72 (0.48 , 1.07) | 0.099 |
|  | 35-44 | 0.46 (0.35 , 0.62) | <0.001 |  | 0.52 (0.36 , 0.75) | 0.001 |
|  | 45-54 | 0.48 (0.35 , 0.65) | <0.001 |  | 0.61 (0.42 , 0.88) | 0.009 |
|  | 55+ | Reference |  |  | Reference |  |
| **Gender** | |  |  |  |  |  |
|  | Female | 0.63 (0.51 , 0.77) | <0.001 |  | 0.50 (0.38 , 0.64) | <0.001 |
|  | Male | Reference |  |  | Reference |  |
| **Race/ethnicity** | |  |  |  |  |  |
|  | Non-Hispanic white | Reference |  |  | Reference |  |
|  | Non-Hispanic black | 0.60 (0.43 , 0.85) | 0.004 |  | 0.49 (0.32 , 0.76) | 0.001 |
|  | Hispanic or Latino | 0.68 (0.41 , 1.12) | 0.129 |  | 0.89 (0.49 , 1.62) | 0.699 |
|  | Other | 0.98 (0.61 , 1.57) | 0.933 |  | 0.85 (0.49 , 1.48) | 0.573 |
| **Marital status** | |  |  |  |  |  |
|  | Never married | 0.49 (0.40 , 0.62) | <0.001 |  | 0.74 (0.55 , 1.01) | 0.058 |
|  | Married | Reference |  |  | Reference |  |
|  | Separated/Divorced/Widowed | 0.52 (0.39 , 0.68) | <0.001 |  | 0.56 (0.40 , 0.79) | 0.001 |
| **Education** | |  |  |  |  |  |
|  | < High school | 0.16 (0.06 , 0.45) | 0.001 |  | 0.19 (0.06 , 0.63) | 0.007 |
|  | High school | 0.41 (0.33 , 0.50) | <0.001 |  | 0.45 (0.34 , 0.58) | <0.001 |
|  | > High school | Reference |  |  | Reference |  |
| **Employment** | |  |  |  |  |  |
|  | Unemployed | Reference |  |  | Reference |  |
|  | Housewife/house husband | 0.81 (0.56 , 1.17) | 0.251 |  | 0.88 (0.56 , 1.39) | 0.583 |
|  | Student | 0.70 (0.41 , 1.18) | 0.182 |  | 1.43 (0.72 , 2.84) | 0.303 |
|  | Employed | 0.91 (0.72 , 1.14) | 0.397 |  | 0.93 (0.69 , 1.25) | 0.622 |
| **Region** | |  |  |  |  |  |
|  | Appalachia South | 0.61 (0.43 , 0.87) | 0.006 |  | 0.76 (0.49 , 1.16) | 0.201 |
|  | Appalachian-North | 0.70 (0.48 , 1.01) | 0.058 |  | 0.70 (0.44 , 1.09) | 0.113 |
|  | Non-Appalachia Rural | 0.86 (0.63 , 1.18) | 0.343 |  | 0.89 (0.61 , 1.29) | 0.527 |
|  | Suburban | 0.81 (0.62 , 1.07) | 0.136 |  | 0.79 (0.57 , 1.09) | 0.150 |
|  | Metro | Reference |  |  | Reference |  |
| **Confidence-state government** | |  |  |  |  |  |
|  | No | 0.32 (0.26 , 0.39) | <0.001 |  | 0.61 (0.44 , 0.83) | 0.002 |
|  | Yes | Reference |  |  | Reference |  |
| **Confidence-federal government** | |  |  |  |  |  |
|  | No | 0.35 (0.28 , 0.42) | <0.001 |  | 0.85 (0.62 , 1.16) | 0.298 |
|  | Yes | Reference |  |  | Reference |  |
| **Confidence- governments around the world** | |  |  |  |  |  |
|  | No | 0.31 (0.24 , 0.4) | <0.001 |  | 0.65 (0.46 , 0.92) | 0.016 |
|  | Yes | Reference |  |  | Reference |  |
| **Knowledge about COVID-19** | | 1.33 (1.25 , 1.40) | <0.001 |  | 1.12 (1.05 , 1.20) | 0.001 |
| **Behavioral adherence** | | 1.59 (1.47 , 1.73) | <0.001 |  | 1.46 (1.31 , 1.62) | <0.001 |
| **Perceived susceptibility** | | 1.30 (1.23 , 1.37) | <0.001 |  | 1.17 (1.09 , 1.27) | <0.001 |
| **Perceived severity** | | 1.26 (1.20 , 1.32) | <0.001 |  | 1.09 (1.01 , 1.17) | 0.021 |
| **Trust in COVID-19 messages from the government** | | 1.14 (1.12 , 1.16) | <0.001 |  | 1.11 (1.08 , 1.13) | <0.001 |
| Abbreviations: OR: odds ratio; CI: confidence interval | | |  |  |  |  |
| Due to small cell sizes participants who identify themselves other than male or female were excluded (n=6) | | | | | | |
